# Functionalization of Polypropylene Films with 2-(Diethylamino)ethyl Methacrylate for Dual Stimuli-Responsive Drug Loading and Release Studies

**DOI:** 10.3390/polym18010068

**Published:** 2025-12-26

**Authors:** José M. Pérez-Larios, Miguel S. Pérez-Garibay, Emilio Bucio

**Affiliations:** Departamento de Química de Radiaciones y Radioquímica, Instituto de Ciencias Nucleares, Universidad Nacional Autónoma de México, Circuito Exterior, Ciudad Universitaria, Mexico City 04510, Mexico; josemanuel.perez@correo.nucleares.unam.mx

**Keywords:** polypropylene, DEAEM, gamma-ray, pH-response, thermoresponsive, ciprofloxacin

## Abstract

This research involved functionalizing polypropylene (PP) films with 2-(Diethylamino)ethyl methacrylate (DEAEM), a monomer that responds to both temperature and pH. For this, Gamma-ray irradiation was used at a dose rate of 11.75 kGy h^−1^, doses ranging from 30 to 100 kGy, and a monomer concentration of 50% (*v*/*v*). The modified films (PP-g-DEAEM) were characterized by thermal analysis, FTIR-ATR, swelling, and contact angle. Confirming that the films were successfully grafted with DEAEM, improving the wettability of the pristine PP films, with a critical pH of 5.6 and a temperature response at 45.7 °C. Subsequently, the films were subjected to ciprofloxacin loading and release, and their in vitro efficacy against the *E. coli* strain was assessed using the Kirby-Bauer method. This work suggests potential applications in biomedical devices; however, further studies are needed.

## 1. Introduction

Polypropylene (PP) is a thermoplastic polymer used in the medical industry, especially for manufacturing medical devices like syringes, vials, catheters, implants, and prostheses, because its linear structure offers chemical resistance, low density, and high mechanical strength [1,2]. PP is a hydrophobic and inert polymer [3,4]. As a result, PP can be readily colonized by biofilm-forming microorganisms [5,6,7,8], leading to various infections. In this context, modifying the polymer’s inherent properties, such as reducing hydrophobicity by adding polar molecules, can enhance its interaction with antibacterial agents [9]. In this sense, stimuli-sensitive polymers, also known as smart polymers, can change their volume in response to slight variations in temperature, pH, electric field, and other stimuli [10]. These materials have been the subject of study for biomedical applications, for example, controlled drug delivery and bioactive surface modification [11,12].

Previous studies have incorporated drugs into PP matrices modified by various techniques, such as plasma treatment [13,14,15,16]. Besides plasma-based methods, other approaches can also add chemical functionality to PP surfaces, such as gamma radiation-induced graft polymerization, which is widely used in biomedicine [17]. The main difference between the two is gamma radiation’s greater penetrating power [18].

Gamma rays are an efficient method that offers several advantages over other methods, such as the lack of additives to initiate the reaction, as well as the rapid and uniform generation of active radical sites, not making temperature a critical factor in the grafting process, and enabling the use of various techniques such as direct and pre-irradiation oxidative methods [19,20]. In this research, the pre-irradiation oxidative method was studied. This method involves irradiating the polymeric matrix in the presence of oxygen, resulting in the formation of peroxides and hydroperoxides that decompose at higher temperatures and act as radical initiators for the grafting process [21,22,23,24]. This method was applied to minimize toxic residues from homopolymerization and to provide precise process control during the grafting reaction [25].

2-(Diethylamino)ethyl methacrylate (DEAEM, Sigma-Aldrich, St. Louis, MO, USA) was studied because it has been reported to exhibit pH- and thermo-responsiveness [26,27]. Previous studies show that adding DMAEM to PP matrices through gamma, electron beam, or UV radiation improves their wettability by increasing surface energy and their ability to form hydrogen bonds or electrostatic interactions [28,29,30].

In this study, DEAEM was grafted onto PP films to improve PP properties, including increased wettability and dual stimulus response, pH, and temperature. PP-g-DEAEM enabled drug loading and release and exhibited antibacterial properties, making it a potential option for medical device applications.

## 2. Materials and Methods

### 2.1. Materials

Isotactic PP films of 1 mm in thickness were obtained from Goodfellow (Huntingdon, England). 2-(Diethylamino)ethyl methacrylate (99%) was obtained from Sigma-Aldrich (St. Louis, MO, USA) and purified by vacuum distillation before use. Toluene and ethanol were from CONQUIMEX (Veracruz, Mexico). Ciprofloxacin was obtained from Sigma-Aldrich (St. Louis, MO, USA) and was used without purification. Hydrochloric acid (36.5–38%) was obtained from Reactivos Meyer (Ciudad de México, Mexico). Citric acid and sodium phosphate, dibasic, anhydrous, were purchased from J.T. Baker (Ciudad de México, Mexico) and used as received. Brain heart infusion broth and Luria broth, both brand BD Difco, as well as Strains *Staphylococcus aureus* (ATCC 25923) and *Escherichia coli* (ATCC 25922) were purchased from Cientifica SENNA (Ciudad de México, Mexico).

### 2.2. Synthesis of PPgDEAEM by Gamma Radiation

PP films were cut into 2.5 cm^2^ pieces and washed with ethanol for 2 h to remove impurities. Then, they were vacuum dried at room temperature for 5 h, and their weight was subsequently recorded (W_0_). Afterwards, PP films were placed into ampoules and exposed to a ^60^Co γ-source (Gammabeam 651 PT, MDSNordion, Ottawa, ON, Canada) in air at room temperature, at a dose rate of 11.75 kGy h^−1^, with doses ranging from 30 to 100 kGy. After irradiation, 5 mL of 50% (*v*/*v*) DEAEM in toluene was added to the ampoules. Then, the ampoules were degassed by repeated freeze–thaw cycles, sealed, and heated to 50 °C for 5 h. Subsequently, the films were removed from the ampoules and washed in ethanol for 24 h to eliminate non-grafted homopolymer. Finally, films were vacuum dried at room temperature for 5 h before recording the final weight (W_g_). The graft percentage was calculated as follows.

(1) $$Graft\left(\%\right)=\frac{W_{g}-W_{0}}{W_{0}}*100$$

where W_g_ is the final weight of the grafted copolymer, and W_0_ is the initial weight of the film PP.

### 2.3. Physicochemical Characterization

#### 2.3.1. Fourier Transform Infrared Spectroscopy with Attenuated Total Reflectance (FTIR-ATR)

Samples of PP film, PP-g-DEAEM (12% grafting), and DEAEM polymer were dried at 60 °C for 24 h before FTIR-ATR analysis, which was performed with a Perkin-Elmer Spectrum 100 (Norwalk, CT, USA) spectrophotometer, performing 16 scans for each using the ATR modulus.

#### 2.3.2. Thermogravimetric Analysis (TGA)

About 10–20 mg of different films, PP, PP-g-DEAEM (12% graft), and DEAEM homopolymer were weighed. Before TGA, samples were maintained at 60 °C for 24 h to ensure maximum moisture removal. Then, samples were placed on the platinum tray of the thermogravimetric analysis equipment TGA Q50 from TA Instruments, New Castle, DE, USA. Experiments were carried out in the temperature range of 25–800 °C under a nitrogen atmosphere, with a heating rate of 10 °C min^−1^.

#### 2.3.3. Differential Scanning Calorimetry (DSC)

About 5–10 mg of PP film, PP-g-DEAEM film (12% grafting), and DEAEM homopolymer were maintained at 60 °C for 24 h to ensure maximum moisture removal. Runs were recorded from 10 to 400 °C at a heating rate of 10 °C min^−1^. Particularly for the PP-g-DEAEM film, a second test was performed to determine the lower critical solution temperature (LCST). A swollen sample of PP-g-DEAEM (overnight) was run from 15 to 80 °C at a rate of 1 °C min^−1^. The LCST was calculated from 30 to 50 °C using the Boltzmann sigmoid function fitting in Origin, as reported previously [31]. All samples were run under a nitrogen atmosphere using a DSC 2010 calorimeter (TA Instruments, USA), starting at room temperature.

#### 2.3.4. Swelling Tests

To determine the swelling limit, PP and PP-g-DEAEM films were initially weighed, then immersed in distilled water and buffer solutions at various pH levels (2–11). At different times, the films were removed from the medium, excess water was wiped off, and the films were reweighed. All swelling experiments were taken in triplicate. The degree of swelling of the samples was calculated using Equation (2).

(2) $$Swelling\left(\%\right)=\frac{W_{s}-W_{d}}{W_{d}}*100$$

W_s_ is the weight of the swollen film at time (t), and W_d_ is the weight of the dry film.

The critical pH was calculated as the inflection point of the swelling limit vs. pH, determined by Boltzmann function fitting.

#### 2.3.5. Contact Angle

The contact angle was measured using a drop shape analyzer, Krüss DSA100 apparatus (Krüss, Matthews, NC, USA). Small drops of distilled water were deposited onto dry films, and the contact angle was measured at room temperature. For each film, the measurements were taken in triplicate.

### 2.4. Ciprofloxacin Loading and Release Studies

#### Ciprofloxacin Loading

The ciprofloxacin loading was performed on approximately 100 mg of PP and PP-g-DEAEM (12% graft) films. The films were placed in vials containing 5 mL of an aqueous ciprofloxacin solution (10 μg/mL) under mechanical stirring at 25 °C. Drug loading was assessed by measuring absorbance at 266 nm with a UV-Vis spectrophotometer (SPECORD^®^ 200 Plus, Analytik, Jena, Germany) at specific intervals until a stable absorbance reading was reached. The concentration of loaded ciprofloxacin from the solution was calculated as follows (3).

(3) $$Drug\;loaded=\left(\frac{Co-Cf}{W}\right)*V$$

where Cf is the final concentration of ciprofloxacin in the solution, and Co corresponds to the initial concentration of ciprofloxacin in the solution. V is the volume of the medium, and W is the weight of the film.

The release of ciprofloxacin was evaluated by immersing the loaded films in vials with 5 mL of phosphate buffer (pH 7.4) at 37 °C. At predetermined intervals, aliquots (3 mL) were collected from the release medium and analyzed by UV spectrophotometry at 266 nm. Once the loading process was complete, the films were removed from the vials and dried for 24 h at room temperature, protected from sunlight. The concentration of drug released was determined using the calibration curve presented in Equation (4).

A = 0.0958C − 0.049 R^2^ = 0.994 (4)

where A is absorbance, and C is the concentration of ciprofloxacin (μg/mL).

### 2.5. Antimicrobial Test

Hinton agar plates were prepared by adjusting the medium’s pH with 1.0 M HCl or 1.0 M NaOH as required, followed by sterilization at 121 °C for 15 min and subsequent cooling to 45 °C before pouring into Petri dishes, which were then incubated at 35 °C for 24 h to ensure sterility. Freeze-dried strains of *Staphylococcus aureus* (ATCC 25923) and *Escherichia coli* (ATCC 25922) were reactivated in brain heart infusion broth or Luria broth for 24 h, after which 100 µL of each culture was inoculated into fresh media and incubated for an additional 7 h; the resulting suspensions were adjusted to a microbial density equivalent to the 0.5 McFarland standard. Bacterial concentrations were determined using the surface extension method after preparing six serial dilutions in isotonic saline, plating 100 µL of the 10^−4^, 10^−5^, and 10^−6^ dilutions onto Hinton–Mueller agar, and incubating for 24 h at 35 °C, resulting in counts of 1.30 × 10^8^ CFU/mL for *E. coli* and 2.5 × 10^8^ CFU/mL for *S. aureus*. For the antimicrobial inhibition assay, each standardized bacterial suspension (0.5 MF) was spread across the surface of Hinton agar plates using a sterile swab. The test films were positioned onto the inoculated agar, and the plates were incubated at 35 °C for 24 h prior to measuring the resulting inhibition zones with a vernier caliper.

## 3. Results

### 3.1. Obtention of PP-g-DEAEM

**Figure 1:**
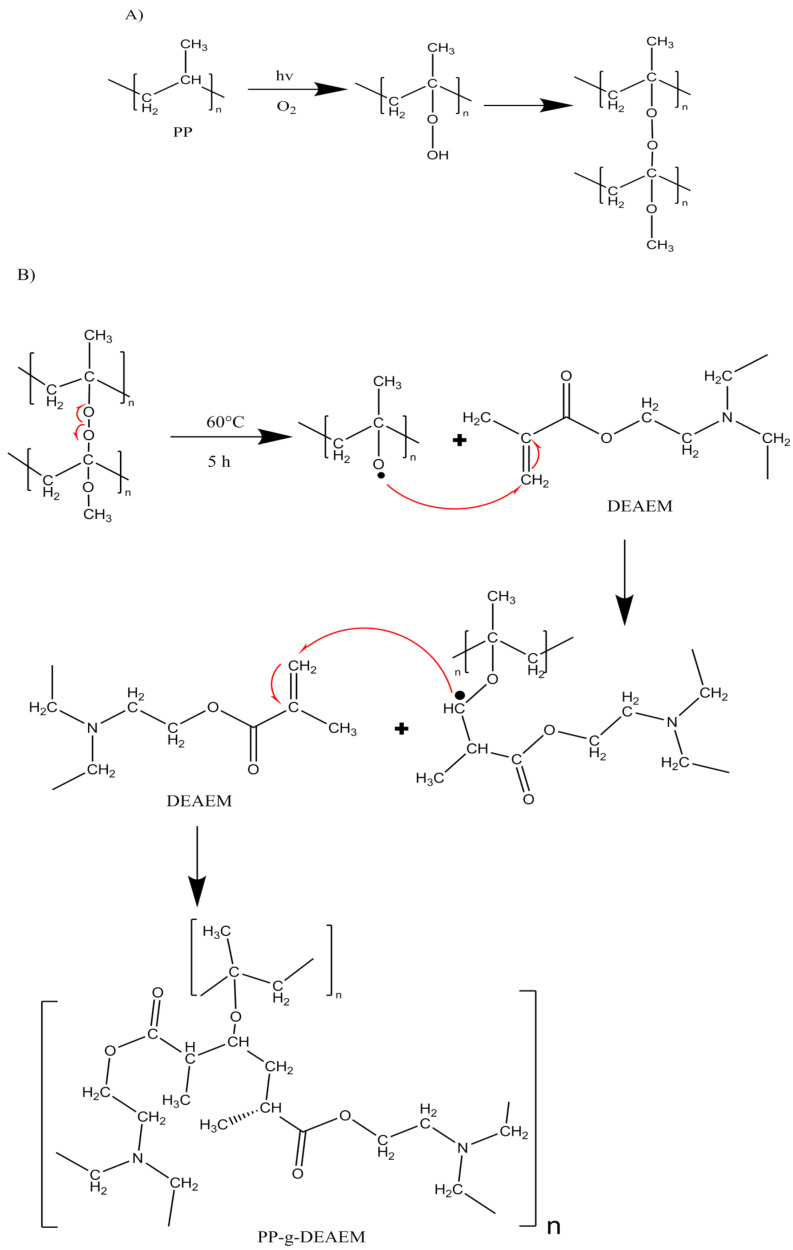
(**A**) Formation of peroxides by gamma radiation in PP. (**B**) Grafting reaction for the synthesis of the copolymer.

**Figure 2:**
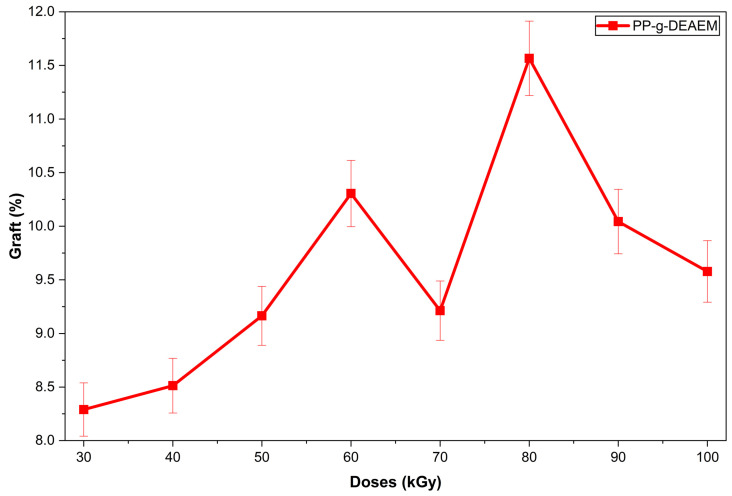
Graph of DEAEM grafting onto PP films at different doses. *n* = 3. Error bars correspond to the standard deviation.

Results of PP-g-DEAEM are shown in Figure 1. Grafting increases with increasing dose, reaching a maximum at 80 kGy. This is attributed to the optimal formation of peroxides at this dose (Figure 1), which react with DEAEM. At doses of 90 kGy or higher (Figure 2), two factors may contribute to a decrease in grafting: (i) the start of PP degradation and excessive peroxide formation in the PP matrix, leading to reactions between them, reducing monomer grafting [32,33,34].

### 3.2. FTIR-ATR Analysis

**Figure 3:**
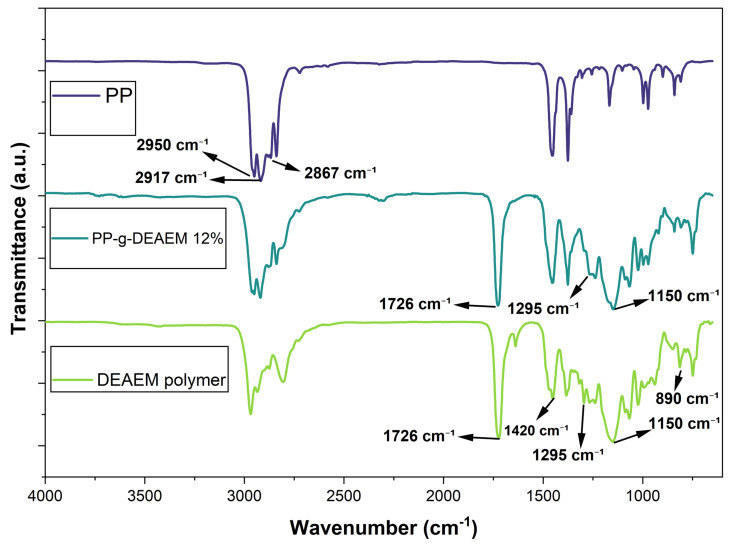
FTIR-ATR spectra of PP, PP-g-DEAEM 12%, and Polymer DEAEM.

The grafting of DEAEM onto PP film was confirmed by FTIR spectroscopy. As shown in Figure 3, the PP film’s spectrum displays characteristic absorption bands related to hydrocarbon chains, such as the asymmetric and symmetric stretching vibrations of the methyl groups at 2950 and 2867 cm^−1^, respectively, as well as the asymmetric and symmetric stretching vibrations of the methyl groups at 2917 cm^−1^ [35].

On the other hand, the spectrum of the DEAEM polymer shows a strong absorption peak at 1726 cm^−1^, attributed to the ester carbonyl group. Bands at 1295 and 1150 cm^−1^ correspond to the symmetric and asymmetric stretching vibrations of this carbonyl group [36]. Additionally, vibrations typical of alkene groups are observed, including a symmetrical stretching vibration at 2975 cm^−1^, a scissoring vibration at 1420 cm^−1^, and a torsional vibration at 890 cm^−1^ [37].

Finally, the spectrum of PP-g-DEAEM displays bands at 1726, 1295, and 1150 cm^−1^, corresponding to the ester, thus confirming the successful grafting of DEAEM onto the PP film.

### 3.3. Thermogravimetric Analysis

**Figure 4:**
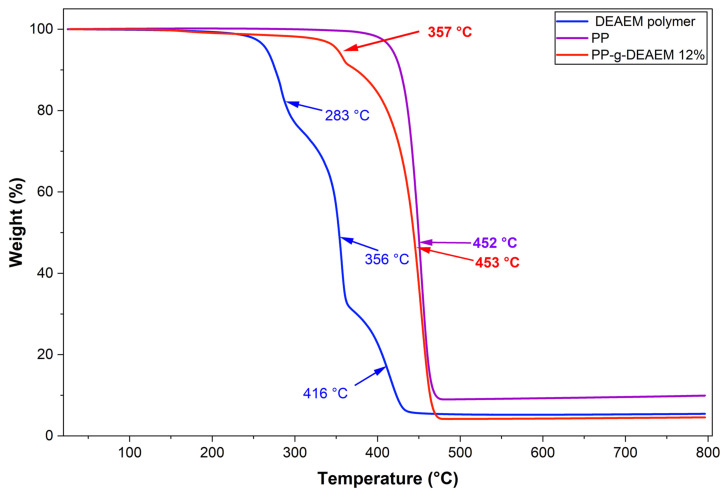
TGA of PP, DEAEM polymer, and PP-g-DEAEM 12%.

Percentage mass loss as a function of temperature was determined by thermogravimetric analysis (TGA). PP, PP-g-DEAEM films, and the DEAEM polymer (Figure 4) were analyzed. The PP film exhibited a 10% weight loss at 427 °C, a decomposition temperature at 452 °C, attributed to the breaking of C–C bonds along the polymer backbone [38], and a char yield of 9.9% at 800 °C.

For the DEAEM polymer, an initial 10% weight loss occurs at 276 °C, followed by three decomposition temperatures at 283, 356, and 416 °C. The first two are ascribed to the removal of diethylaminoethyl groups, while the third one involves the release of CO_2_ and CO and includes carbonization processes [39,40]. The char yield at 800 °C was approximately 5.5%.

The PP-g-DEAEM film exhibited intermediate thermal behavior, with a 10% weight loss at 374 °C. Two decomposition temperatures were identified: one at 453 °C, corresponding to the breakdown of the polypropylene backbone, and the other at 357 °C, attributed to the degradation of the grafted DEAEM segments.

### 3.4. DSC Analysis

**Figure 5:**
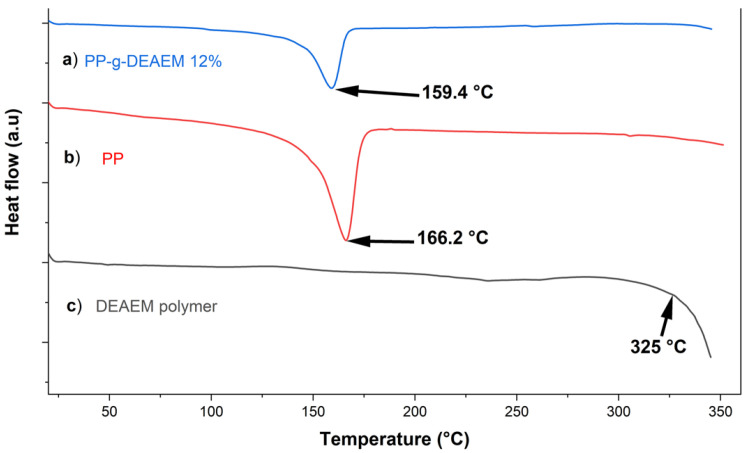
DSC analysis. (**a**) PP-g-DEAEM 12%. (**b**) PP. (**c**) DEAEM polymer.

DSC Figure 5 was performed to evaluate the thermal transitions of the films. The melting point (T_m_) of PP was determined to be about 166.2 °C. In comparison, the PP-g-DEAEM was slightly lower at 159.4 °C, indicating that the thermal stability of polypropylene was not significantly affected by the DEAEM graft. The DEAEM polymer under the conditions studied did not exhibit a melting point or a glass transition temperature; however, it began to decompose at temperatures up to 325 °C.

**Figure 6:**
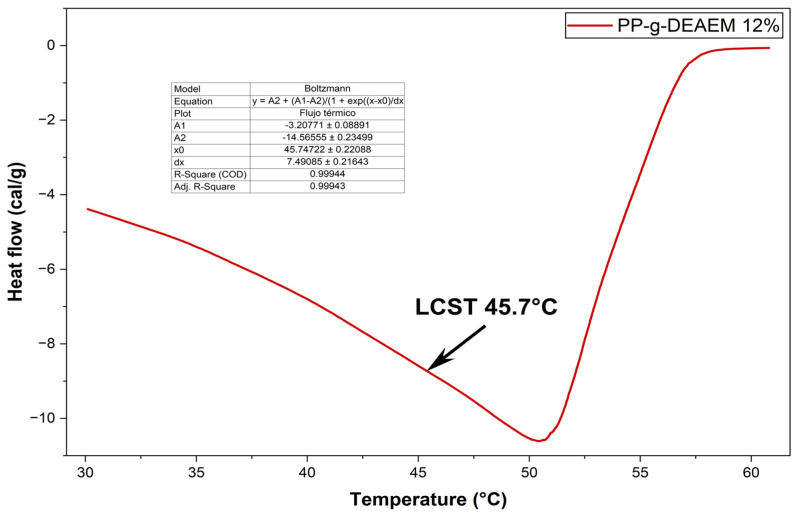
LCST of PP-DEAM 12%.

Finally, the swollen PP-g-DEAEM shows an LCST at 45.7 °C (Figure 6). Using the Boltzmann sigmoid function fit allows determining the LCST based on heat flow versus temperature. When the system reaches its LCST, a phase transition occurs within a narrow temperature range (45.7 °C); at this point, it fits as a sigmoidal drop in heat flow. Previous studies have reported values ranging from 14 to 72 °C [41,42]; these differences are due to factors like copolymer composition, molecular weight, solution concentration, and pH [41].

### 3.5. Swelling and Contact Angle

**Figure 7:**
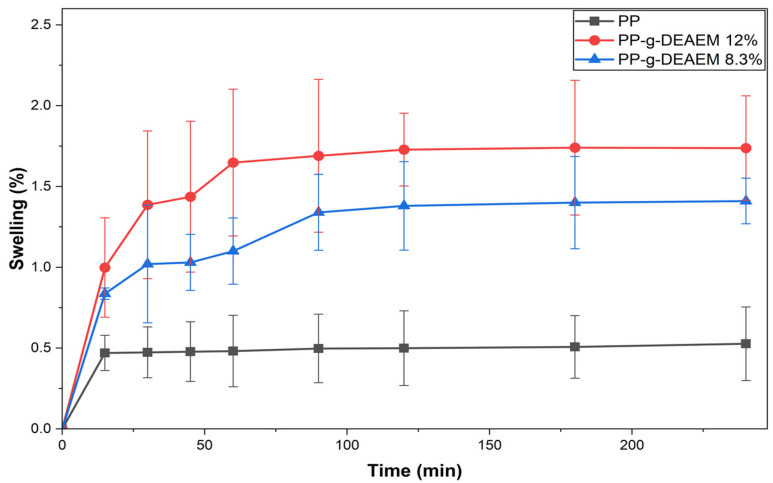
Swelling index in distilled water at room temperature for films PP and PP-g-DEAEM—12% and 8.3%.

Figure 7 shows the swelling results in distilled water. PP films exhibited almost no swelling (0.5%), as expected due to their hydrophobic nature [43]. In contrast, PP-g-DEAEM reached a maximum swelling of 1.74% and 1.3% for grafts of 12% and 8.3%, respectively, after 2 h, which is attributed to hydrogen bonding between the carbonyl group of DEAEM and water.

**Figure 8:**
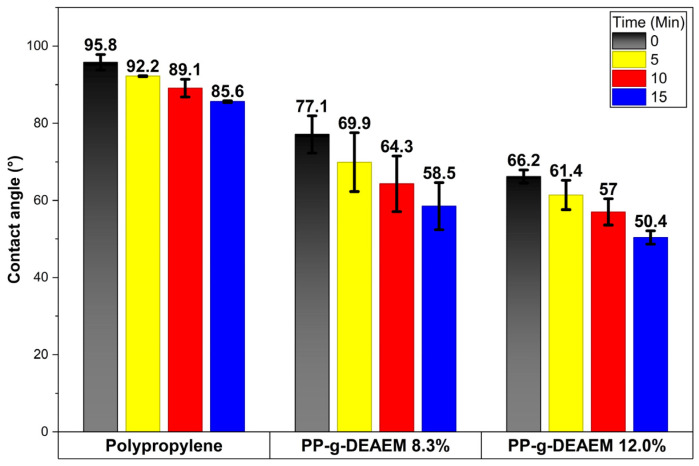
Contact angle behavior PP-g-DEAEM (12%) at 0, 5, 10, and 15 min. *n* = 3. Error bars correspond to the standard deviation.

Contact angle results are presented in Figure 8. The PP film at 0 min exhibited a contact angle of 95.8°, which decreased to 85.6° after 15 min due to its hydrophobic nature [44]. These values fall within the range previously reported [45,46].

Conversely, PP-g-DEAEM showed a notable decrease in contact angle, indicating enhanced hydrophilicity. At time 0 min, the contact angles were 77.1 and 66.2°, while at 15 min, the contact angles were 58.5 and 50.4° for grafting degrees of 8.3 and 12%, respectively. The reduction in the contact angle is due to the addition of DEAEM, which can form hydrogen bonds with water.

### 3.6. Determination of Critical pH

**Figure 9:**
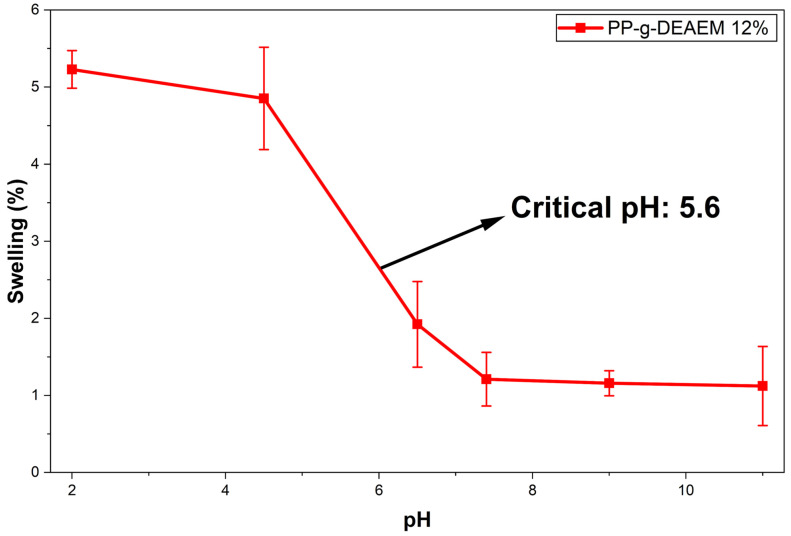
Swelling profile as a function of the pH of PP-g-DEAEM 12%. *n* = 3 measures. Error bars correspond to the standard deviation.

**Figure 10:**
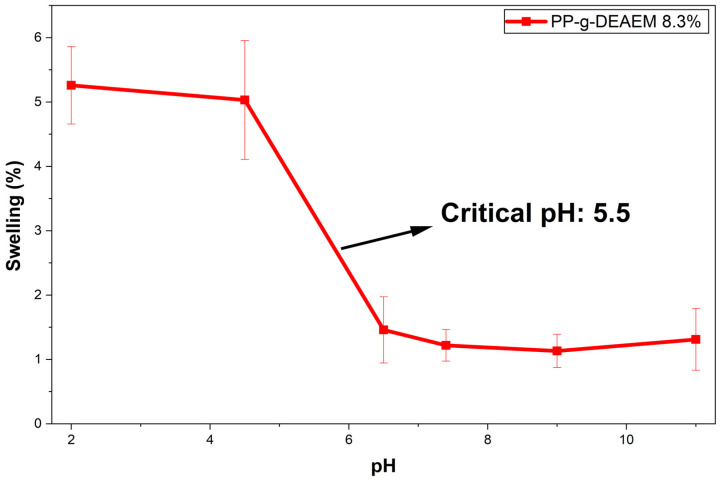
Swelling profile as a function of the pH of PP-g-DEAEM 8.3%. *n* = 3 measures. Error bars correspond to the standard deviation.

The pH reaction experiment was conducted over a broad pH range (2–11). As shown in Figure 9 and Figure 10. The swelling of PP-g-DEAEM increases at acidic pH due to the protonation of tertiary amines, resulting in a swollen state [47,48,49]. As the pH increases above the critical pH of 5.6, swelling decreases, leading the material to enter a collapsed state, which indicates that it loses the hydrophilicity gained through DEAEM grafting [50]. It is worth noting that the swelling profile of the material with 8.3% grafting closely resembles that of 12%, indicating that, within this range, the degree of grafting does not significantly affect the response to pH.

In a previous study [30], the addition of acrylic acid (AAc) and DEAEM significantly affects the pH response of DEAEM. When both monomers are used on PP, AAc introduces carboxyl groups whose charge varies with pH. In acidic conditions, the -COOH groups of AAc are not ionized, so the system remains collapsed even if DEAEM is protonated. At intermediate pH (~5), some of the AAc is ionized as -COO^−^, and DEAEM is protonated, allowing the formation of polyelectrolyte complexes between the -COO^−^ and –NH^+^ groups of DEAEM, which increases swelling of the material. Finally, at high pH, AAc is fully ionized, and the repulsion between the -COO^−^ groups greatly enhances swelling [30]. Comparing AAc and DEAEM, AAc results in a more pronounced, non-linear pH response, primarily affecting DEAEM’s behavior.

### 3.7. Loading and Release of Ciprofloxacin

**Figure 11:**
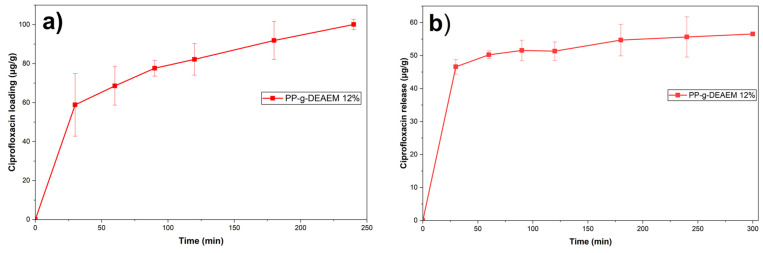
(**a**) Ciprofloxacin loading curve of PP-g-DEAEM 12%. (**b**) Ciprofloxacin release curve of PP-g-DEAEM 12%. *n* = 3 measures. Error bars correspond to the standard deviation.

PP films did not load ciprofloxacin as expected, attributed to insufficient interactions between PP and ciprofloxacin. On the other hand, Figure 11a shows the results for the loading of ciprofloxacin in PP-g-DEAEM film, reaching a maximum loading of 100 μg/g at 4 h. The loading is mainly due to hydrogen-bond interactions between ciprofloxacin and the DEAEM chains.

**Figure 12:**
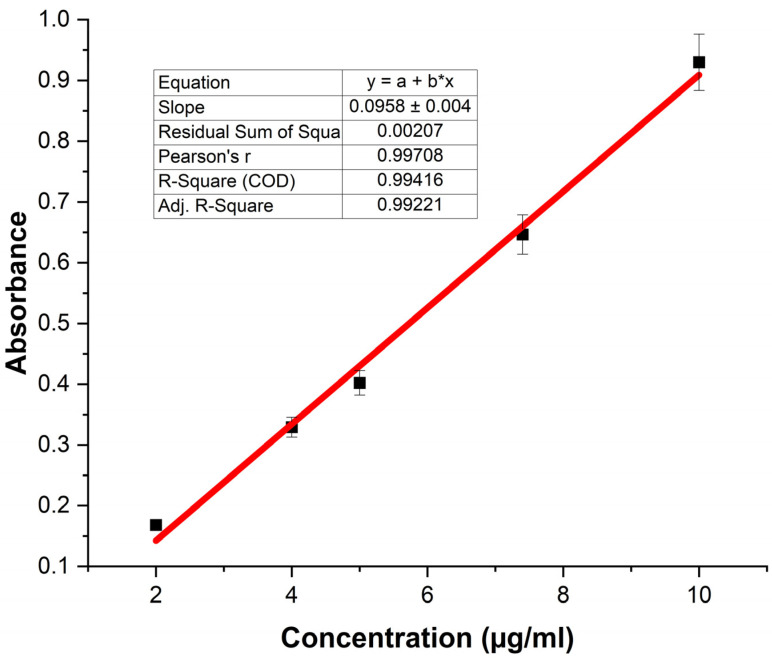
Calibration curve of ciprofloxacin. *n* = 3 measures. Error bars correspond to the standard deviation.

The release results are shown in Figure 11b. Approximately 55% of the ciprofloxacin incorporated in the PP-g-DEAEM was released. The limited release is attributed to the same interactions that led to ciprofloxacin loading. Additionally, the release studies were conducted below the LCST (45.7 °C), resulting in films that remained swollen and retained the drug. However, this must be confirmed in future studies that vary in temperature and pH. Release data indicated that the best-fit release kinetics was the Peppas-Sahlin model (R^2^ = 0.9987), suggesting that the kinetics are governed by the coupling of two mechanisms: Fickian diffusion, in which the drug diffuses through the swollen polymer matrix, and polymer relaxation [51]. Ciprofloxacin concentrations used for both loading and release analyses were calculated using a UV–Vis calibration curve of ciprofloxacin (Figure 12).

### 3.8. Results of Antimicrobial Test

**Figure 13:**
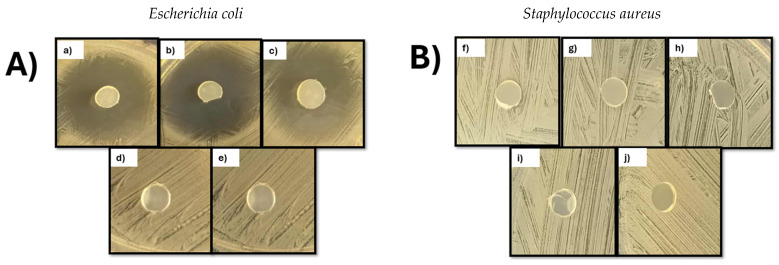
Results of antimicrobial test (**A**) *E. coli* and (**B**) *S. aureus*. PP-g-DEAEM 12% film loaded with ciprofloxacin (**a**–**c**,**f**–**h**). Controls PP-g-DEAEM 12% (**d**,**i**) and PP film (**e**,**j**).

The antimicrobial activity of the films is shown in Figure 13. PP and PP-g-DEAEM films were tested. The ciprofloxacin-loaded PP-g-DEAEM films exhibited inhibition halos of 25 mm against *E. coli*, indicating an intermediate susceptibility category (I) according to the CLSI guidelines [52]. The term intermediate indicates that the microorganism is neither completely susceptible nor resistant; its susceptibility to the antibacterial agent depends on its concentration and, therefore, requires higher doses to achieve effective inhibition [53]. *E. coli* has been reported to be responsible for about 50% of urinary catheter-related infections [54]. In this regard, PP-g-DEAEM can be a potential option for medical devices used to treat urinary infections. For *S. aureus*, no inhibition halos were observed with any film. This may be due to ciprofloxacin’s interaction with its target enzymes, a higher MIC for *S. aureus* (compared to *E. coli*), the amount of drug loaded, the release kinetics of ciprofloxacin, and differences in cell wall permeability [55,56].

## 4. Discussion

The increase in grafting degree, from 8.3% to 11.6%, is due to a higher production of primary radicals in the membrane under irradiation conditions. However, with further dose increases, a drop in grafting degree was seen at 90 kGy. This behavior is explained by the fact that excessive irradiation doses lead to free-radical recombination and polymer matrix degradation, thereby limiting grafting efficiency.

In this regard, the increased number of grafted DEAEM chains led to a higher density of tertiary amine groups on the PP surface, thus directly improving hydrophilicity and water absorption. This is consistent with the smaller contact angles observed for the film with 12% grafting and its greater water swelling compared to films with 8.3% grafting. A higher concentration of DEAEM groups increases the number of sites capable of forming hydrogen bonds with water and being protonated at acidic pH, thereby facilitating matrix hydration. Similar results were obtained [29,30], where the addition of DEAEM significantly improved the hydrophilicity of PP.

The DEAEM polymer is a polyelectrolyte with an approximate pKa of 7.3, whose tertiary amines can be protonated or deprotonated depending on the pH of the medium. As the pH increases, the amines become deprotonated, decreasing the material’s affinity for water. This behavior is associated with the formation of hydrogen bonds between DEAEM and water molecules at low temperatures, which weaken as the temperature rises because the polymer becomes more hydrophobic.

The increased hydrophilicity and swelling capacity also improve drug-loading performance. In this study, the PP-g-DEAEM film (12%) showed approximately 100 μg/g of ciprofloxacin uptake, due to the presence of tertiary amine groups that interact with the drug via hydrogen bonds and electrostatic interactions. On the other hand, ciprofloxacin has two ionizable groups—a carboxyl group (pKa_1_ = 5.90) and a piperazinyl group (pKa_2_ = 8.89)—allowing it to adopt cationic, zwitterionic, or anionic forms depending on the pH. This variation directly affects its interaction with the polymer system and the potentiometric response, promoting the formation of hydrogen bonds and electrostatic attractions in distilled water. Drug release was tested at pH 7.4. Although the films collapse at this pH, at 37 °C, PP-g-DEAEM films swell enough to enable partial drug release. However, limited swelling restricts total drug release and may even cause the film to reabsorb the drug. Similar results have been seen in DEAEM-based hydrogels and nanoparticles, where DEAEM influences the loading of different drugs [57,58,59].

Regarding the release experiments, pH 7.4 and 37 °C were selected as standard physiological conditions, commonly used to assess the baseline release behavior of new drug-delivery materials. These conditions serve as a reference point for comparison with other studies and help ensure that the system functions in a controlled, widely accepted environment.

Since release kinetics occur within 4 h, this initial release can cause rapid antibiotic distribution in the agar and, consequently, a visible zone of inhibition during the assay [60,61]. The methodology used in this work serves as a qualitative indicator of antimicrobial activity and, on its own, does not reflect the release duration [61]. To quantitatively connect the release profile to antimicrobial activity, additional assays must be performed.

Only *S. aureus* and *E. coli* were selected for microbiological tests because these two species are widely recognized as representative models of Gram-positive and Gram-negative bacteria, respectively. In addition to confirming that ciprofloxacin was loaded, the objective was to detect inhibition halos.

The final biomedical use of the modified polypropylene films remains undefined, as this stage is a preliminary study focused on developing and understanding pH-responsive materials capable of loading and releasing ciprofloxacin. Once more studies are conducted with different variables, the final application may become clearer.

## 5. Conclusions

The grafting of the DEAEM monomer onto polypropylene (PP) films via gamma radiation was successful. However, the grafting efficiency using this method did not surpass 12%, with the optimal result observed at 80 kGy. Various characterization techniques, including FTIR-ATR, TGA, and DSC, confirmed modifications to the pristine PP films. Contact angle measurements indicated that the PP films exhibited reduced hydrophobicity, with the contact angle of both pristine PP and PP-g-DEAEM (12%) decreasing by approximately 35° after 15 min. Swelling assessments at different pH values demonstrated PP-g-DEAEM’s responsiveness to pH changes, with a critical pH of 5.6. The incorporation of DEAEM facilitated the loading of approximately 100 µg/g of ciprofloxacin, which was subsequently released. Antimicrobial testing validated the drug loading, as evidenced by 25 mm inhibition zones against *E. coli*. This investigation remains preliminary; further studies are required, including evaluations of mechanical properties, cytocompatibility, atomic force microscopy, drug loading with alternative drugs, and exploration of other methods or modifications to enhance monomer grafting efficiency. Nonetheless, PP-g-DEAEM demonstrates potential for the production of antimicrobial sanitary disposables.

## Data Availability

The original contributions presented in this study are included in the article. Further inquiries can be directed to the corresponding authors.
